# Mesenchymal Stem Cell-Derived Exosomal miRNA-222-3p Increases Th1/Th2 Ratio and Promotes Apoptosis of Acute Myeloid Leukemia Cells

**DOI:** 10.1155/2023/4024887

**Published:** 2023-08-16

**Authors:** Yuan Yuan, Shengfen Tan, Huanhuan Wang, Junfeng Zhu, Jiajia Li, Pingping Zhang, Meng Wang, Feng Zhang

**Affiliations:** ^1^Department of Hematology, First Affiliated Hospital of Bengbu Medical College, Bengbu, Anhui 233004, China; ^2^Department of Hematology, Bengbu Medical College, Bengbu, Anhui 233004, China

## Abstract

Interferon regulatory factor 2 (IRF2) participates in the differentiation of immune T cells. Bone marrow mesenchymal stem cell (BM-MSC)-derived exosomes can secret mRNA, miRNAs, and proteins to regulate tumor microenvironment. The present study focused on the miRNA/IRF2 axis in regulating Th1/Th2 ratio and cell apoptosis in acute myeloid leukemia (AML). The flow cytometry analysis was performed to examine the Th1/Th2 ratio and AML apoptosis *in vivo* and *in vitro*. The contents of Interferon *γ* (IFN-*γ*) and Interleukin-4 (IL-4) were measured using enzyme-linked immunosorbent assay. StarBase was used to predict the potential binding site between miR-222-3p and the 3′ untranslated region of IRF2. Luciferase reporter assay was applied for validating the combination of miR-222-3p and IRF2. BM-MSC exosomes were successfully isolated. BM-MSC exosomes increased Th1/Th2 ratio and promoted apoptosis of AML cells. Further analysis showed that IRF2 was targeted by miR-222-3p. Overexpression of miR-222-3p promoted Th1/Th2 ratio and AML cell apoptosis. IRF2 partially reversed the effect that is exerted by miR-222-3p on Th1/Th2 ratio and AML cell apoptosis. Overexpression of miR-222-3p promoted Th1/Th2 ratio and caspase 3 expression *in vivo*. To sum up, miR-222-3p promotes Th1/Th2 ratio and AML cell apoptosis by regulating IRF2 expression, which provided crucial targets for the treatment of AML.

## 1. Introduction

Acute myeloid leukemia (AML) is a common malignant tumor in the hematopoietic system [1]. The feature of AML is the malignant clonal proliferation of highly heterogeneous hematopoietic stem cells and precursor cells [2]. Although the development of new agents and the improvement of treatment have improved the survival rate of AML patients, the recurrence after treatment is still a major challenge to the survival of patients [3, 4]. Thus, it is of great significance to further understand the AML pathogenesis, which may provide novel therapy strategies to improve the overall survival of patients.

Mesenchymal stem cells (MSCs) with the capability of self-renewal and multi-lineage differentiation can differentiate into various cell types, including chondrocytes, adipocytes, osteoblasts, and myoblasts, which has been applied for various disease treatments [5]. MSCs are one of the main cell components in the microenvironment of bone marrow growth. Under physiological conditions, MSCs can regulate bone marrow hematopoietic function by interacting with hematopoietic stem cells [6, 7]. MSCs are known as the stepping stones of the microenvironment of hematopoietic stem cells, and hematopoietic insufficiency is a sign for AML. Evidence has manifested that the function of MSCs from AML patients has changed [8, 9]. Corradi et al., proved that MSCs derived from AML patients have decreased *in vitro* viability and can promote the survival of AML cells, revealing that MSCs are critically involved in AML [10]. Among them, because bone marrow mesenchymal stromal cells (BM-MSC) and adipose stem cells are easily accessible and have no ethical issues, they are considered to be the most suitable cells in clinical trials [11]. Evidence manifested that BM-MSC can participate in modulating hematopoietic function by controlling the equilibrium of differentiation of hematopoietic stem cells/progenitor cells. It is used in a variety of malignant hematological tumors, including AML [12].

Exosomes are cystic vesicles wrapped in nanometer-scale cell membrane secreted by cells through budding, which is rich in mRNA, miRNAs, and protein [13, 14]. Exosomes could inject the contents of mRNA, miRNAs, and protein into target cells through fusion with target cells for the modulation of cell proliferation, apoptosis, and migration [15]. MicroRNA is a new short non-coding RNA molecule. Increasing evidence has proved that BM-MSC exosomes regulate cancer development by modulating miRNA expression in the tumors and the surrounding cells [14, 16]. Moreover, exosomal miRNAs derived from BM-MSC are also indicated to involve in regulating AML pathogenesis [17]. Our previous studies have proved that the interferon regulatory factor 2 (IRF2)/Inositol polyphosphate 4-phosphatase type II (INPP4B) promotes AML progression by facilitating AML cell proliferative capability and inhibiting apoptosis [18, 19]. MiRNAs can modulate gene expression mainly through the complementary binding of miRNA and the 3′-UTR of the target mRNAs to suppress mRNA translation or promote mRNA degradation [20]. In the current research, hsa-miR-222-3p has the potential to bind to IRF2 mRNA, which is predicted by the starBase. In addition, it has been proved that IRF2 can promote Th2 cell immune response and inhibit Th1 cell immune response by regulating the balance of Th1/Th2 cell ratio [11, 12], suggesting that IRF2 plays an important role in the differentiation of immune T cells. However, the function of the miR-222-3p/IRF2 axis on AML development remains unclear. Thus, this study aimed to investigate the mechanism of the miR-222-3p/IRF2 axis on AML development.

## 2. Materials and Methods

### 2.1. Patients and Specimens

Twenty AML patients and healthy donors were collected from the First Affiliated Hospital of Bengbu Medical College in 2021. All patients signed the informed consents. Bone marrow tissues were collected in a heparin anticoagulation tube and mixed well. Then the bone marrow fluid was transferred to a new centrifuge tube, and the same amount of 0.01 M Phosphate-buffered saline (PBS) was added and mixed, followed by centrifugation at 1500 rpm for 10 minutes. The bone marrow fluid was transferred to the upper layer of the Ficoll separation solution and then centrifuged for 30 minutes at 2500 rpm. Cells were gently aspirated at the tunica albuginea interface and transferred to a new centrifuge tube. Later, they were diluted with 0.01 M PBS and then centrifugated twice at 1500 rpm for 10 minutes. After collecting the precipitate, we added the cell culture medium and resuspend the cells, and then cells were put in a constant temperature incubator. This study was approved by the Ethics Committee of the First Affiliated Hospital of Bengbu Medical College.

### 2.2. Exosome Extraction

BM-MSC were incubated in serum-free medium and then centrifuged for 10 minutes at 5000 rpm. Then the medium was filtrated by a 0.22 *μ*m filter, and moderate Eppendorf solution was added into the supernatant, gently mixed, and then cultured at 4°C for 1 hour. Subsequently, the mixed solution received centrifugation for 15 minutes at 12,000 rpm at 4°C. The precipitates containing the exosomes were obtained and resuspended in PS buffer (500 *μ*l, 4478359, Invitrogen). The exosomes were stored at −80°C for further analysis.

### 2.3. Transmission Electron Microscopy

Transmission electron microscopy (TEM) was applied for examining the traits of BM-MSC exosomes. Briefly, exosome suspension (5 *μ*l) was pipetted onto the copper grids and maintained for 20 minutes at room temperature. Subsequently, the exosomes were washed with PBS for 5 minutes. Next, 1% glutaraldehyde was used to fix the exosomes for 5 minutes, and ddH_2_O was used for washing for 2 minutes with 10 times. Next, 4% uranium dioxo was added and acetate for 5 minutes. The sample was air-dried for 2 hours and analyzed at 120 kV using TEM (HT7700; HITACHI, Tokyo, Japan).

### 2.4. Nanoparticle Tracking Analysis

The size of exosomes was analyzed by nanoparticle tracking analysis (NTA). Briefly, the exosomes were isolated and then diluted in pure water to generate 20–40 particles/view. Then the exosome sizes were examined using dynamic light scattering (ZetaView PMX 110, Particle Metrix, Germany).

### 2.5. Cell Culture and Treatment

The BM-MSC was isolated and then incubated in Fatal bovine serun (FBS)-free medium. Subsequently, BM-MSC exosomes were isolated and incubated with miR-222-3p mimics/inhibitors for 24 hours. Then the HL60 cells were treated with BM-MSC exosomes. The Th1/Th2 ratio and the apoptosis of HL60 were assayed after treatment for 48 hours. The sequences of SiRNA of IRF2 were as follows: si-IRF2-1, sense sequence: 5′-AGUUAAGCACAUCAAGCAAGA-3′; antisense sequence: 5′-UUGCUUGAUGUGCUUAACUUU-3′; si-IRF2-2, sense sequence: 5′-GGUGAACAUCAUAGUUGUAGG-3′; antisense sequence: 5′-UACAACUAUGAUGUUCACCGU-3′; si-IRF2-3, sense sequence: 5′-GGUCCUGACUUCAACUAUAAA-3′; antisense sequence: 5′-UAUAGUUGAAGUCAGGACCGC-3′; miR-222-3p mimics, sense sequence: AGCUACAUCUGGCUACUGGGU; antisense sequence: ACCCAGUAGCCAGAUGUAGCU; and miR-222-3p inhibitor: ACCCAGUAGCCAGAUGUAGCU.

### 2.6. Quantitative Real-Time PCR

Total RNA isolation was performed via TRIzol Reagent (KCDR2001, Cronda). The collected RNAs were reversely transcribed by PrimeScript RT Reagent Kit (KCDR2001, Cronda). Then the Polymerase chain reaction (qPCR) was conducted on LightCycler® 480II (Roche) by using SYBR Premix DimerEraser (Q711-02, Vazyme). The relative gene expression was analyzed with the 2^−*ΔΔ*Ct^ methods. *β*-actin and U6 served as the internal reference for mRNA and miRNA, respectively. Sequences of primers are as follows: IRF2, forward: 5′-AGTACGGTGAACATCATAGTTG-3′, reverse: 5′-TGTCGGTAGTTTCGCTTT-3′; caspase 3, forward: 5′-ACAGCACCTGGTTACTATTC-3′, reverse: 5′- CAGTTCTTTCGTGAGCAT-3′; *β*-actin, forward: 5′-GTCCCTCACCCTCCCAAAAG-3′, reverse: 5′-GCTGCCTCAACACCTCAACCC-3′; miR-222-3p reverse primer: 5′-CTCAACTGGTGTCGTGGAGTCGGCAATTCAGTTGAGACCCAGTA-3′; miR-222-3p, forward: 5′-GCACTAGCTACATCTGGC-3′; and U6, forward: 5′-CTCGCTTCGGCAGCACA-3′, reverse: 5′-AACGCTTCACGAATTTGCGT-3′.

### 2.7. Western Blot

The cultured cells or tissues were harvested and used to extract total proteins by RIPA lysis (KCD-M1013, Cronda). The concentration of the total protein from each treatment was detected using Bicinchoninic acid assay (BL521A, Biosharp). Then equal protein was used to load on 12% Sodium dodecyl sulfate polyacrylamide gel electropheresis. The proteins were transferred to Polyvinylidene fluoride membranes after separation. Subsequently, the membranes were sealed by 5% non-fat milk for 2 hours and incubated with primary antibodies anti-IRF2 (1 : 1000, abcam, ab124744), anti-CD6 (1 : 2000, abcam, ab109217), anti-CD9 (1 : 5000, abcam, ab236630), anti-TSG101 (1 : 5000, abcam, ab125011), anti-caspase 3 (1 : 2000, abcam, ab32351), and anti-actin (1 : 2000, Serivicebio, GB12001) for one night at 4°C. The membrane was cultured by secondary antibody (1 : 4,000, BL003A, biosharp) for 1 hour at 37°C. Finally, the protein band was imaged by enhanced chemiluminescence (ECL) in a darkroom. Image J was used to detect the relative grey density.

### 2.8. Luciferase Reporter Assay

The 3′ UTR of IRF2 with wild type (WT) binding site or mutant (MUT) binding site was inserted into the pmirGLO reporter vectors. Then the pmirGLO vector and miR-222-3p mimics were co-transfected into cells by a Lipofectamine™ 2000 kit (11668-027, Invitrogen). A luciferase reporter kit (Promega) was applied for the detection of the relative luciferase reporter activity.

### 2.9. Flow Cytometry

The Th1/Th2 ratio and apoptosis were detected by flow cytometry. For the CD4+ T cell differentiation, CD4+ T cells were blocked with 2% FBS for 30 minutes and then incubated with anti-CD4-APC for 30 minutes. Then 2% paraformaldehyde was applied to fix cells. For apoptosis analysis, AML cells with different treatment were collected and digested with 0.25% trypsin. Then PBS was used to wash the cells twice, and 100 *μ*L of binding buffer was added for incubation. Next, cells received the treatment of AnnexinV-FITC and PI (HS-SJ069, Hasenbio) at ambient temperature in dark for 5 minutes. The cells were examined by FACSVerse (BD Biosciences), and results were analyzed by FlowJo software (BD).

### 2.10. AML Mice Model

Four to five weeks male Server combined immune-deficiency (SCID) Beige mice weighted at 15–19 g were supplied by Comparative Medicine Center of Yangzhou University and feed in a Specific pathogen free condition. For AML model establishment, mice were injected with cyclophosphamide intraperitoneally (100 mg/kg). Four days later, the injection of 3 × 10^6^ HL60 cells into SCID mice was performed via tail vein. Then the mice were treated with 10 *μ*g/25 *μ*l PBS BM-MSC exosomes with miR-222-3p mimics/inhibitors. The mouse peripheral blood samples were isolated for 28 days with BM-MSC exosomes treatment, which was used for further study. All experimental procedures were approved by the Institutional Animal Care and Use Committee of the First Affiliated Hospital of Bengbu Medical College.

### 2.11. ELISA

The content of Interleukin-4 (IL-4) (E-EL-H0101c, Elabscience) and Interferon *γ* (IFN-*γ*) (E-EL-H0108c, Elabscience) was detected by Enzyme linked immunosorbent assay (ELISA). Briefly, the ELISA kit was taken out and then kept for 15 minutes. Then samples and standards were supplemented to corresponding wells and cultured for 3 hours. Later, the liquid in the ELISA plate was discarded, and eluent was added for washing the plate three times. Biotin-labeled working solution was added for incubation for 1 hour. Then the liquid in the plate was discarded, and the eluent was added to wash for three times. The enzyme binding working solution was added and cultured for half an hour, and 100 *μ*l of color developing solution was supplemented for incubation in dark at room temperature for 15 minutes. 100 *μ*l of stop solution was added and mixed. The absorbance value was detected within 30 minutes. A standard curve was drawn, and the sample concentration was calculated.

### 2.12. Statistical Analysis

All statistical analyses were analyzed by the SPSS 20.0. Data were presented as mean ± standard deviation (SD) with at least for three repeats. The unpaired Student's t-test was utilized for comparing two groups, and the one-way ANOVA was utilized for comparing with more than two groups, followed by Tukey's *post hoc* analysis. *P* values <0.05 were considered as statistically significant.

## 3. Results

### 3.1. BM-MSC Exosomes Increase Th1/Th2 Ratio and Promote AML Cell Apoptosis

**Figure 1:**
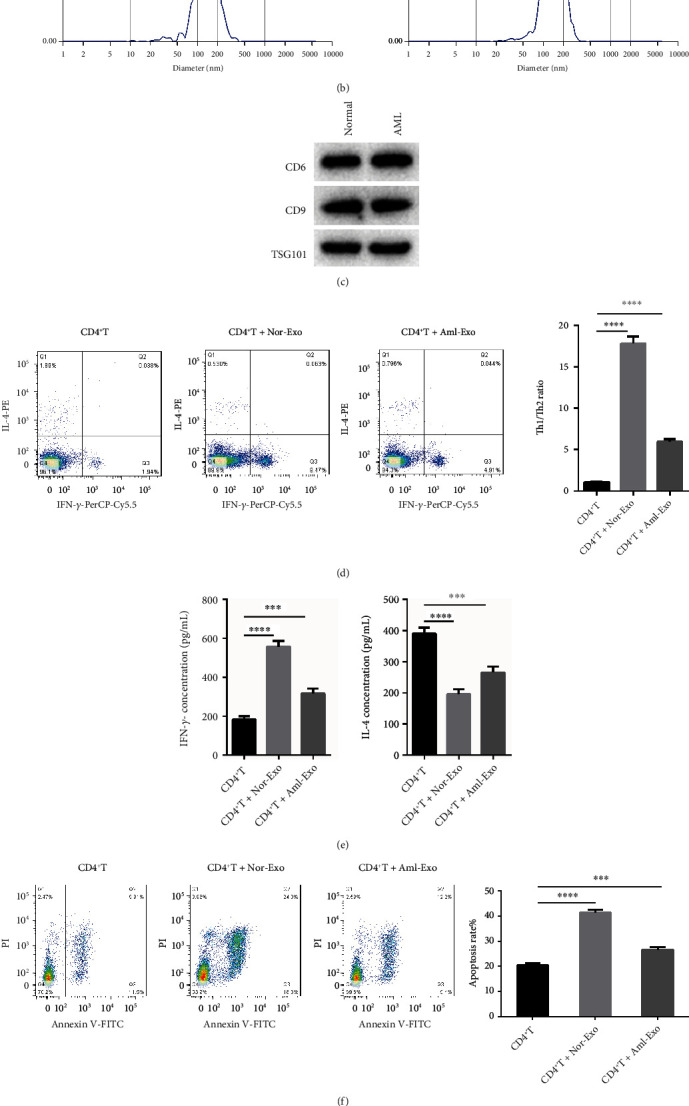
BM-MSC exosomes increase Th1/Th2 ratio and promote AML cell apoptosis. (a) BM-MSC exosomes were isolated from patients with AML and healthy control individuals, and the phenotype of exosomes was detected by TEM. (b) NTA assay was applied to examine the exosome size (normal: 139.8 nm and AML: 140.5 nm). (c) The protein expression of exosome markers CD6, CD9, and Tsg101 was determined by western blot in the normal group and the AML group. (d) The Th1/Th2 ratio was detected by flow cytometry affected by BM-MSC exosomes from both AML patients and healthy individuals. (e) ELISA was used to detect the contents of IFN-*γ* and IL-4 affected by BM-MSC exosomes from AML patients and healthy individuals. (f) The apoptosis of AML cell was detected by flow cytometry treatment with BM-MSC exosomes from AML patients and healthy individuals. (g) The caspase 3 protein level changes affected by BM-MSC exosomes from AML patients and healthy individuals. *β*-actin acts as control. ∗*p* < 0.05, ∗∗∗*p* < 0.001, ∗∗∗∗*p* < 0.0001.

To know how exosomes functioned on differentiation of immune T cells and AML cell apoptosis, BM-MSC exosomes were isolated from AML patients and healthy individuals. As shown in Figure 1(a), TEM assay showed that exosomes have an obvious membrane boundary under the electron microscope, showing a saucer or cup-like structure of peak sizes at 139.8 and 140.5 nm (Figure 1(b)). In addition, we found that in the exosomes, exosome marker (CD6, CD9, and Tsg101) expression was abundant (Figure 1(c)), which suggested that the BM-MSC exosomes were successfully isolated in the AML patients or healthy individuals. Then, the CD4+ T cells were treated by BM-MSC exosomes. The results indicated that the Th1/Th2 ratio was significantly increased when treatment with BM-MSC exosomes from both AML patients and healthy individuals. Further analysis showed that Th1/Th2 rate was reduced in exosomes of AML patients than those in healthy individuals (Figure 1(d)). Furthermore, we found IFN-*γ* content was significantly increased when treatment with BM-MSC exosomes from both AML patients and normal individuals, while IFN-*γ* content in exosomes from AML patients was lower than that from healthy individuals. IL-4 content had the opposite outcome compared with the changes of IFN-*γ* (Figure 1(e)). Moreover, we found that AML cell apoptosis was promoted by CD4+ T cells treatment with BM-MSC exosomes compared with CD4+ T group. However, AML cell apoptosis was inhibited by exosomes from AML patients than those from normal individuals (Figure 1(f)). These results were also confirmed for the caspase 3 expression changes (Figure 1(g)). In short, we confirmed that BM-MSC exosomes elevated Th1/Th2 ratio and promoted apoptosis of AML cells.

### 3.2. IRF2 Is Targeted by miR-222-3p

**Figure 2:**
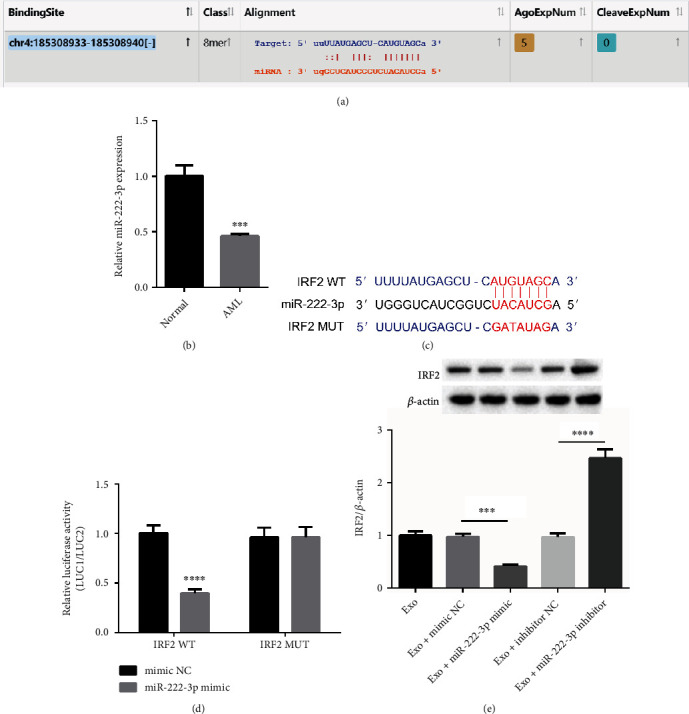
IRF2 is targeted by miR-222-3p. (a) StarBase was used for the prediction of miRNAs targeting IRF2 and potential binding sites between miR-222-3p and IRF2 3′ UTR. (b) The expression of miR-222-3p in BM-MSC exosomes from patients with AML and the exosomes from healthy control individuals was detected by RT-qPCR. (c) The binding sites between miR-222-3p and IRF2 3′ UTR. (d) Dual luciferase reporter assay was applied to examine the relative luciferase activity of IRF2 WT or IRF2 MUT in the mimic NC group and the miR-222-3p mimic group. (e) The protein expression of IRF2 affected by miR-222-3p was determined by western blot in the exosome group, the exosome + mimic NC group, the exosome + miR-222-3p mimic group, the exosome + inhibitor NC group, and the exosome + miR-222-3p inhibitor group. *β*-actin acts as control. ∗∗∗*p* < 0.001, ∗∗∗∗*p* < 0.0001.

The critical role of IRF2 in AML development has been revealed. In order to explore the regulatory mechanism of IRF2, starBase (https://starbase.sysu.edu.cn/) was applied to predict IRF2 miRNAs targets. We found potential binding site between IRF2 3′ UTR and miR-222-3p (Figure 2(a)). Furthermore, miR-222-3p expression was significantly downregulated in the BM-MSC exosomes from AML patients in comparison with the exosomes from healthy individuals (Figure 2(b)), suggesting miR-222-3p was critically participated in AML development. Furthermore, we found the significant decrease in the relative luciferase reporter activity when being co-transfected with miR-222-3p mimics and IRF2-pmiRGLO WT vector in comparison with the NC group. On contrary, the relative luciferase activity of IRF2-pmiRGLO Mut vectors presented no significant change by miR-222-3p mimics compared with NC group (Figures 2(c) and 2(d)). In addition, we found that the expression of IRF2 was greatly downregulated after miR-222-3p upregulation while elevated after miR-222-3p downregulation (Figure 2(e)). Overall, IRF2 can be targeted by miR-222-3p.

### 3.3. MiR-222-3p Overexpression Promotes Th1/Th2 Ratio and AML Apoptosis

**Figure 3:**
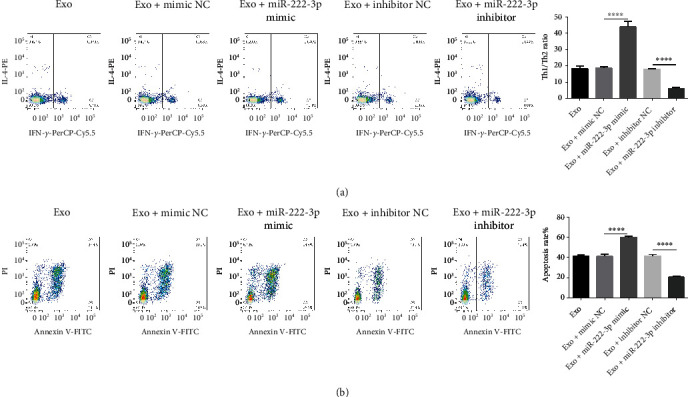
Overexpression of BM-MSC exosomal miR-222-3p promotes Th1/Th2 ratio and AML apoptosis. (a) The Th1/Th2 ratio was detected by flow cytometry in the exosome group, the exosome + mimic NC group, the exosome + miR-222-3p mimic group, the exosome + inhibitor NC group, and the exosome + miR-222-3p inhibitor group. (b) The apoptosis of HL60 cells was measured using flow cytometry in the above five groups. ∗∗∗∗*p* < 0.0001.

For the sake of investigating miR-222-3p function on Th1/Th2 ratio and AML apoptosis, HL60 cells were treated with BM-MSC exosomes with miR-222-3p overexpression or suppression. Flow cytometry manifested that exosomes and miR-222-3p upregulation could increase Th1/Th2 ratio, while miR-222-3p inhibitor decreased the Th1/Th2 ratio (Figure 3(a)). Furthermore, BM-MSC exosomes with miR-222-3p upregulation could markedly promoted HL60 cell apoptosis, while BM-MSC exosomes with miR-222-3p inhibitor suppressed HL60 cell apoptosis compared with their corresponding NC control group (Figure 3(b)). Thus, overexpression of BM-MSC exosomal miR-222-3p facilitated Th1/Th2 ratio and AML apoptosis.

### 3.4. IRF2 Partially Reverses the Effects Exerted by miR-222-3p on Th1/Th2 Ratio and AML Apoptosis

**Figure 4:**
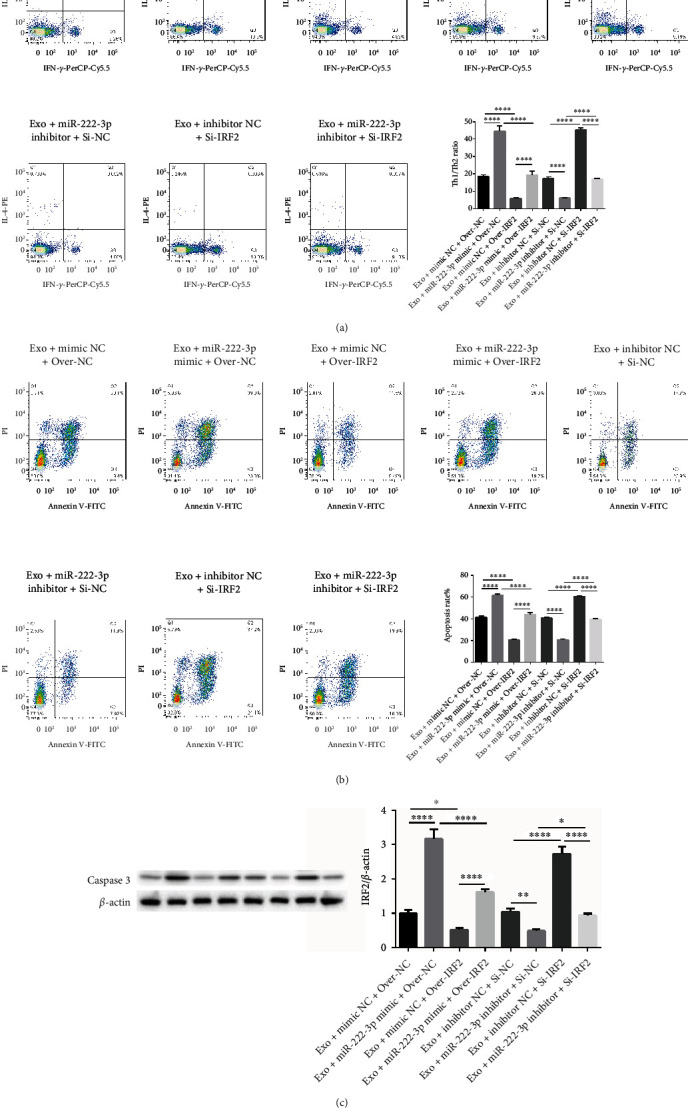
IRF2 partially reverses the effect exerted by miR-222-3p on Th1/Th2 ratio and AML apoptosis. (a and b) The Th1/Th2 ratio and AML apoptosis were both detected by flow cytometry in the exosome + mimic NC + Over-NC group, the exosome + miR-222-3p mimic + over-NC group, the exosome + mimic NC + over-IRF2 group, the exosome + miR-222-3p mimic + over-IRF2 group, the exosome + inhibitor NC + Si-NC group, the exosome + miR-222-3p inhibitor + Si-NC group, the exosome + inhibitor NC + Si-IRF2 group, and the exosome + miR-222-3p inhibitor + Si-IRF2 group. (c) Western blot was used to detect the expression of caspase 3 in the above eight groups. *β*-actin acts as control. ∗*p* < 0.05, ∗∗∗∗*p* < 0.0001.

In order to know whether the function of IRF2 on Th1/Th2 ratio and AML apoptosis regulated by miR-222-3p, recovery experiment was performed. As shown in Figure 4(a), the elevated Th1/Th2 ratio by BM-MSC exosomes and miR-222-3p mimics as well as the decrease by exosomes with miR-222-3p inhibitor could be reversed by treatment with overexpressing IRF2 plasmid or SiRNA of IRF2 compared with the NC group (Figure 4(a)). Furthermore, the elevated apoptosis by BM-MSC exosomes and miR-222-3p mimics or the decrease by BM-MSC exosomes with miR-222-3p inhibitor could be decreased or increased by treatment with overexpressing IRF2 plasmid or SiRNA of IRF2 (Figure 4(b)). In addition, we found that exosomes with miR-222-3p upregulation could increase caspase 3 expression, while miR-222-3p suppression reduced the caspase 3 expression compared with their corresponding NC control group (Figure 4(c)). On contrary, the expression of caspase 3 could be partially reversed by treatment with overexpressing IRF2 plasmid or SiRNA of IRF2 (Figure 4(c)). These results indicated that IRF2 partially reversed the effects exerted by miR-222-3p on Th1/Th2 ratio and AML apoptosis.

### 3.5. Overexpression of miR-222-3p Facilitates Th1/Th2 Ratio and Caspase 3 Expression *In Vivo*

**Figure 5:**
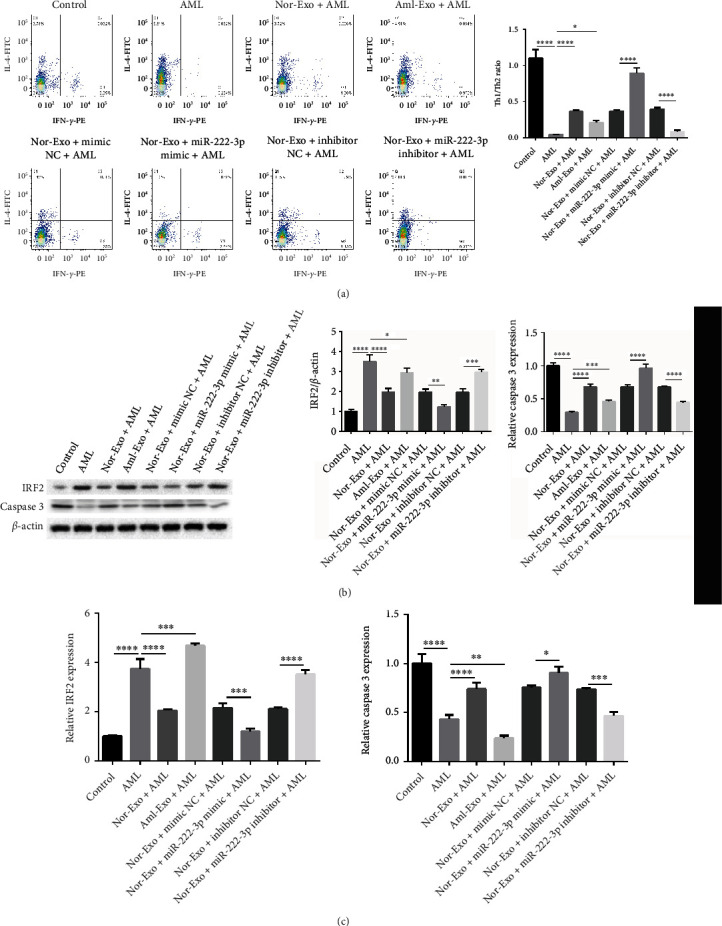
Overexpression of BM-MSC exosomal miR-222-3p promotes Th1/Th2 ratio and caspase 3 expression *in vivo*. (a) Flow cytometry analysis was performed to analyze the Th1/Th2 ratio *in vivo* in the control group, the AML group, the Nor-exosome + AML group, and the Aml-exosome + AML group, the Nor-exosome + mimic NC + AML group, the Nor-exosome + miR-222-3p mimic + AML group, the Nor-exosome + inhibitor NC + AML group, and the Nor-exosome + miR-222-3p inhibitor + AML group. (b and c) The mRNA and protein expression of IRF2 and caspase 3 expression in the above eight groups were measured by western blot and RT-qPCR, respectively. ∗*p* < 0.05, ∗∗*p* < 0.01, ∗∗∗*p* < 0.001, ∗∗∗∗*p* < 0.0001.

To further explore the effect of miR-222-3p on Th1/Th2 ratio and caspase 3 expression, AML model was established in SCID mice. Flow cytometry indicated that the Th1/Th2 ratio was reduced in the AML group. However, Th1/Th2 ratio was significantly elevated when treatment with BM-MSC exosomes compared with AML group. In addition, the Th1/Th2 ratio was greatly increased by the addition of exosomes and miR-222-3p mimics but decreasing by exosomes and miR-222-3p downregulation in comparison with their corresponding controls (Figure 5(a)). Moreover, IRF2 expression upregulated in the AML group both at mRNA and protein level. However, we found significant decrease in IFR2 expression when treated with BM-MSC exosomes compared with AML group. Additionally, IRF2 level presented great decrease after treatment with exosomes with miR-222-3p mimics while increased when treatment with exosomes with miR-222-3p downregulation in comparison with their corresponding control (Figures 5(b) and 5(c)). On the contrary, the expression of caspase 3 showed the opposite results compared with the expression of IRF2 with different treatment (Figures 5(b) and 5(c)). Thus, overexpression of BM-MSC exosomal miR-222-3p accelerated Th1/Th2 ratio and caspase 3 expression *in vivo*.

## 4. Discussion

AML is a highly heterogeneous clonal disease caused by acquired myeloid cell malignant proliferation [21]. In recent years, with the advancement in chemotherapy and supportive therapy, the survival rate of AML patients has been significantly improved [22]. However, 20–30% of patients still relapse with merely 40–50% 5-year overall survival [23]. Therefore, in-depth studies on the molecular mechanism in AML are still required. Herein, we confirmed BM-MSC exosomal miR-222-3p promoted Th1/Th2 ratio and AML apoptosis by regulating IRF2, which leads to inhibition of AML development.

The pathogenesis of AML is a multi-step process. The disordered gene modulation, cell differentiation, proliferation as well as apoptosis eventually result in hematopoietic stem and progenitor cell malignant transformation [24]. As the cytogenetics and molecular biology research continuously deepen, the pathogenesis of AML has been further studied, and the role of more genes in AML has been explained, providing more potential possibilities in the early warning, prognostic analysis, and targeted therapy of AML [25]. In this study, IRF2 could promote AML development. IRF2 belongs to the IRF protein family, which was identified as the modulator of the type I interferon system in the beginning [26]. IRF protein family is involved in immune regulation, cell proliferation, lymphocyte differentiation, and is a key factor in tumor occurrence [27]. Bone marrow mesenchymal stem cells (BMSCs) are family members of adult stem cells possessing the ability for multi-differentiation and show the potential in tissue engineering. Accumulating studies confirm BM-MSC exosomes or their carriers played important roles in the AML progression. Such as Zhang et al. indicated that exosomal miR-425-5p from BM-MSCs exerts suppressive effect on the proliferation, metastasis, and promotive effect on apoptosis by interacting with Wilms tumor 1-associated protein in AML [28]. Lyu et al. showed that BM-MSC exosomes facilitate the growth, invasion as well as the chemoresistance by upregulating S100A4 expression in AML cells [29]. In our study, IRF2 was targeted by miR-222-3p. Further analysis indicated that miR-222-3p level was decreased in exosomes from AML patients in comparison with healthy individuals. These results indicated that IRF2 promoted AML progression regulated by miR-222-3p. IRF2 is reported to facilitate cell proliferation and suppress apoptosis of nasopharyngeal carcinoma through AKT pathway [30]. IRF2 modulates cell survival of liver cancer via regulating *β*-catenin [31]. Importantly, our previous studies have proved that the IRF2/INPP4B axis promotes AML progression by promoting the proliferation and survival of AML cells and inhibiting cell apoptosis [18, 19]. These studies further supported our findings.

miRNA is a small non-coding nucleotide single-stranded RNA with a size of 19–25 nt, which plays an important regulatory role by specifically binding to the binding site of the target gene. Increasing evidence confirm miR-222-3p exerts the vital function on assorted diseases. Gan et al. demonstrated that miR-222-3p suppressed osteogenic differentiation of BMSCs through Insulin-like growth factor 1/extracellular regulated protein kinases pathway [32]. Gao et al. indicated that downregulation of miR-222-3p promoted the migration, invasion, and recruitment of Endothelial progenitor cells by activating ADIPOR1-induced 2-amino-3-methyl-4-ketopentanoic acid signaling pathway [33]. And several studies also proved that miR-222-3p acts as potential biomarkers in the tumor development. Such as miR-222-3p is related to metastasis in renal clear cell carcinoma, and plasma exosomal miR-222-3p may be the biomarker in papillary thyroid carcinomas [34, 35]. This study proved that overexpression of BM-MSC exosomal miR-222-3p facilitated AML cell apoptosis via elevating caspase 3 expression, which indicated that BM-MSC exosomal miR-222-3p could reduce AML occurrence and development.

T helper (Th) cells play a key role in the network of T cell immune system [36]. Researches manifest that unbalanced Th1/Th2 participated in different cancer progression [37]. Saikosaponin A suppresses the development of breast cancer via regulating Th1/Th2 balance [38]. Ibrutinib may shape the chronic lymphocytic leukemia T cell profile by regulating the Th2/Th1 ratio [39]. Th1/Th2 shift may be conducive to the inhibition on immune system in AML [40]. It has been proved that IRF2 can promote Th2 cell immune response and inhibit Th1 cell immune response by regulating the balance of Th1/Th2 cell ratio [11, 12]. These findings reveal that IRF2 is critically implicated in immune T cell differentiation. In our previous study, Th1/Th2 rate in the blood of AML patients significantly decreased, and IRF2 can significantly reduce the ratio of normal and AML patients-derived CD4+ T cells differentiated into Th1/Th2 cells [41]. Recently, Zhang et al. indicated that miR-222-3p may be the promising biomarker for thyroid cancer prognosis and it could participate in the regulation of CD4+ T differentiation [42]. In the present study, we demonstrated that BM-MSC exosomes decreased Th1/Th2 ratio and inhibited apoptosis of AML cells. Further analysis indicated that overexpression of BM-MSC exosomal miR-222-3p promotes Th1/Th2 ratio. Furthermore, IRF2 could partially reverse the effect exerted by BM-MSC exosomal miR-222-3p on Th1/Th2 ratio.

AML model was established in SCID mice, and we found that the Th1/Th2 ratio was increased by BM-MSC exosomes and miR-222-3p mimics while decreased by exosomes and miR-222-3p inhibitor. Moreover, IRF2 expression was negatively correlated with miR-222-3p. Caspase 3 expression showed the similar trend with of Th1/Th2 ratio. These results further confirmed the inhibitory function of BM-MSC exosomal miR-222-3p on AML progression *in vivo*.

Taken together, our study demonstrated that BM-MSC exosomal miR-222-3p promoted Th1/Th2 ratio and AML apoptosis by regulating IRF2, thereby inhibiting AML development. However, the limitation of this study is that there is no further study on the signaling pathways that may participate in the development of AML, which will be the focus of future research.

## 5. Conclusion

In this study, BM-MSC exosomal miR-222-3p promotes Th1/Th2 ratio and AML apoptosis by regulating IRF2 expression. These findings might provide clues in better understanding of the mechanisms of AML and also provide crucial targets for the treatment of AML.

## Data Availability

The datasets during and/or analyzed during the current study are available from the corresponding author on reasonable request.
